# Supplementary material for: Molecular origins of exciton condensation in van der Waals heterostructure bilayers
Source: Chem Sci. 2024 Nov 20;15(48):20371–8. doi: 10.1039/d4sc04149f (PMC11578111; doi:10.1039/d4sc04149f)
Supplement: SC-015-D4SC04149F-s001 [file SC-015-D4SC04149F-s001.pdf]

Supplementary Information (SI) for Chemical Science.
This journal is © The Royal Society of Chemistry 2024

Supporting Information: Molecular Origins of Exciton Condensation in Van der Waals Heterostructure Bilayers

Lillian I. Payne Torres, Anna O. Schouten, and David A. Mazziotti*

Department of Chemistry and The James Franck Institute, The University of Chicago, Chicago, IL 60637

E-mail: damazz@uchicago.edu

Table 1: 2×2 $MoSe_2$-WSe_2

Atom	X	Y	Z
Mo	0.00000	1.91869	7.10596
Mo	-1.66163	4.79673	7.10596
Mo	3.32327	1.91869	7.10596
Mo	1.66163	4.79673	7.10596
W	1.66163	0.95935	0.00000
W	0.00000	3.83738	0.00000
W	4.98491	0.95935	0.00000
W	3.32327	3.83738	0.00000
Se	0.00000	1.91869	1.68443
Se	-1.66163	4.79673	1.68443
Se	3.32327	1.91869	1.68443
Se	1.66163	4.79673	1.68443
Se	1.66163	0.95935	5.42989
Se	0.00000	3.83738	5.42989
Se	4.98491	0.95935	5.42989
Se	3.32327	3.83738	5.42989
Se	1.66163	0.95935	8.78170
Se	0.00000	3.83738	8.78170
Se	4.98491	0.95935	8.78170
Se	3.32327	3.83738	8.78170
Se	-1.66163	0.95935	5.42989
Se	-1.66163	0.95935	8.78170
Se	-3.32327	3.83738	5.42989
Se	-3.32327	3.83738	8.78170
Se	-1.66163	6.71542	5.42989
Se	-1.66163	6.71542	8.78170
Se	1.66163	6.71542	5.42989
Se	1.66163	6.71542	8.78170
Se	1.66163	-0.95935	-1.68443
Se	1.66163	-0.95935	1.68443

Atom	X	Y	Z
Se	0.00000	1.91869	-1.68443
Se	3.32327	1.91869	-1.68443
Se	-1.66163	4.79673	-1.68443
Se	1.66163	4.79673	-1.68443
Se	4.98491	-0.95935	-1.68443
Se	4.98491	-0.95935	1.68443
Se	6.64654	1.91869	-1.68443
Se	6.64654	1.91869	1.68443
Se	4.98491	4.79673	-1.68443
Se	4.98491	4.79673	1.68443
H	-3.07009	4.52731	1.21139
H	4.35981	-0.21659	4.71819
H	5.45321	-0.39541	8.30690
H	-1.03654	-0.21659	4.71819
H	-2.12994	-0.39541	8.30690
H	-4.02911	4.96670	4.71819
H	-4.73068	4.10920	8.30690
H	-2.99258	6.76204	4.71819
H	-2.60074	7.79836	8.30690
H	0.33069	6.76204	4.71819
H	0.72253	7.79836	8.30690
H	2.99204	-1.00327	-2.39731
H	2.59918	-2.04440	1.21139
H	-2.36488	3.66653	-2.39731
H	6.31531	-1.00327	-2.39731
H	5.92245	-2.04440	1.21139
H	7.34978	0.78849	-2.39731
H	8.05500	1.64928	1.21139
H	5.68815	3.66653	-2.39731
H	6.39336	4.52731	1.21139

Table 2: 3×3 $MoSe_2$-WSe_2

Atom	X	Y	Z
Mo	0.00000	1.91869	54.32685
Mo	-1.66163	4.79673	54.32685
Mo	-3.32327	7.67476	54.32685
Mo	3.32327	1.91869	54.32685
Mo	1.66163	4.79673	54.32685
Mo	0.00000	7.67476	54.32685
Mo	6.64654	1.91869	54.32685
Mo	4.98491	4.79673	54.32685
Mo	3.32327	7.67476	54.32685
W	1.66163	0.95935	61.43281
W	0.00000	3.83738	61.43281
W	-1.66163	6.71542	61.43281
W	4.98491	0.95935	61.43281
W	3.32327	3.83738	61.43281
W	1.66163	6.71542	61.43281
W	8.30818	0.95935	61.43281
W	6.64654	3.83738	61.43281
W	4.98491	6.71542	61.43281
Se	0.00000	1.91869	59.74838
Se	-1.66163	4.79673	59.74838
Se	-3.32327	7.67476	59.74838
Se	3.32327	1.91869	59.74838
Se	1.66163	4.79673	59.74838
Se	0.00000	7.67476	59.74838
Se	6.64654	1.91869	59.74838
Se	4.98491	4.79673	59.74838
Se	3.32327	7.67476	59.74838
Se	1.66163	0.95935	56.00292
Se	0.00000	3.83738	56.00292
Se	-1.66163	6.71542	56.00292
Se	4.98491	0.95935	56.00292
Se	3.32327	3.83738	56.00292
Se	1.66163	6.71542	56.00292
Se	8.30818	0.95935	56.00292
Se	6.64654	3.83738	56.00292
Se	4.98491	6.71542	56.00292
Se	1.66163	0.95935	52.65111
Se	0.00000	3.83738	52.65111
Se	-1.66163	6.71542	52.65111
Se	4.98491	0.95935	52.65111
Se	3.32327	3.83738	52.65111
Se	1.66163	6.71542	52.65111
Se	8.30818	0.95935	52.65111
Se	6.64654	3.83738	52.65111
Se	4.98491	6.71542	52.65111
Se	-1.66163	0.95935	52.65111
Se	-1.66163	0.95935	56.00292
Se	-3.32327	3.83738	52.65111
Se	-3.32327	3.83738	56.00292
Se	-4.98491	6.71542	52.65111
Se	-4.98491	6.71542	56.00292
Se	-3.32327	9.59346	52.65111
Se	-3.32327	9.59346	56.00292

Atom	X	Y	Z
Se	0.00000	9.59346	52.65111
Se	0.00000	9.59346	56.00292
Se	3.32327	9.59346	52.65111
Se	3.32327	9.59346	56.00292
Se	1.66163	-0.95935	59.74838
Se	1.66163	-0.95935	63.11724
Se	0.00000	1.91869	63.11724
Se	3.32327	1.91869	63.11724
Se	-1.66163	4.79673	63.11724
Se	1.66163	4.79673	63.11724
Se	-3.32327	7.67476	63.11724
Se	0.00000	7.67476	63.11724
Se	4.98491	-0.95935	59.74838
Se	4.98491	-0.95935	63.11724
Se	6.64654	1.91869	63.11724
Se	4.98491	4.79673	63.11724
Se	3.32327	7.67476	63.11724
Se	8.30818	-0.95935	59.74838
Se	8.30818	-0.95935	63.11724
Se	9.96981	1.91869	59.74838
Se	9.96981	1.91869	63.11724
Se	8.30818	4.79673	59.74838
Se	8.30818	4.79673	63.11724
Se	6.64654	7.67476	59.74838
Se	6.64654	7.67476	63.11724
H	-4.02651	6.54457	59.03550
H	8.77658	-0.39540	55.52819
H	7.68302	-0.21643	51.93918
H	-1.17892	1.23804	51.24775
H	-3.06908	1.23107	55.52819
H	-2.84056	4.11608	51.24775
H	-4.73072	4.10910	55.52819
H	-4.50219	6.99411	51.24775
H	-5.45331	5.36067	55.52819
H	-1.99245	9.63994	51.93918
H	-2.38423	10.67648	55.52819
H	0.00000	9.03607	51.24775
H	0.93904	10.67648	55.52819
H	3.32327	9.03607	51.24775
H	2.38423	10.67648	55.52819
H	1.66163	-0.39836	58.34646
H	2.59918	-2.04440	62.64420
H	-4.73173	7.40535	62.64420
H	4.98491	-0.39836	58.34646
H	5.92245	-2.04440	62.64420
H	9.63858	-1.00327	59.03550
H	9.24572	-2.04440	62.64420
H	10.67305	0.78849	59.03550
H	11.37827	1.64928	62.64420
H	7.82235	4.51623	58.34646
H	7.82235	4.51623	64.51916
H	6.16071	7.39427	58.34646
H	7.11745	9.02923	62.64420

Table 3: 2×2 MoS_2-WS_2

Atom	X	Y	Z
W	1.59000	0.91799	3.12500
W	0.00000	3.67195	3.12500
W	4.77000	0.91799	3.12500
W	3.18000	3.67195	3.12500
S	0.00000	1.83597	4.68750
S	-1.59000	4.58993	4.68750
S	3.18000	1.83597	4.68750
S	1.59000	4.58993	4.68750
S	0.00000	1.83597	1.56250
S	-1.59000	4.58993	1.56250
S	3.18000	1.83597	1.56250
S	1.59000	4.58993	1.56250
S	1.59000	-0.91799	1.56250
S	1.59000	-0.91799	4.68750
S	4.77000	-0.91799	1.56250
S	4.77000	-0.91799	4.68750
S	6.36000	1.83597	1.56250
S	6.36000	1.83597	4.68750
S	4.77000	4.58993	1.56250
S	4.77000	4.58993	4.68750
S	1.58030	6.38762	11.44518
S	1.58030	6.38762	8.43288
Mo	1.58030	4.56262	9.93898
S	3.16080	3.65012	11.44518
S	-1.58070	6.38762	11.44518
S	-1.58070	6.38762	8.43288
S	3.16080	3.65012	8.43288
Mo	3.16080	1.82512	9.93898
Mo	-1.58070	4.56262	9.93898
S	4.74130	0.91262	8.43288

Atom	X	Y	Z
S	-0.00020	3.65012	11.44518
S	4.74130	0.91262	11.44518
S	-0.00020	3.65012	8.43288
Mo	-0.00020	1.82512	9.93898
S	1.58030	0.91262	11.44518
S	-3.16120	3.65012	8.43288
S	-3.16120	3.65012	11.44518
S	1.58030	0.91262	8.43288
S	-1.58070	0.91262	11.44518
S	-1.58070	0.91262	8.43288
H	-2.85281	4.33418	4.25218
H	-2.23721	3.57691	0.92648
H	0.38910	-0.97198	0.92648
H	0.73710	-1.88374	4.25218
H	3.56910	-0.97198	0.92648
H	3.91710	-1.88374	4.25218
H	5.93929	1.59307	0.29222
H	6.76992	3.05747	4.25219
H	4.34929	4.34703	0.29222
H	6.03281	4.33418	4.25218
H	0.71885	7.34179	11.00125
H	1.58030	5.92112	7.15539
H	-0.71925	7.34179	11.00125
H	-0.37719	6.45572	7.80318
H	4.19852	-0.16370	7.80318
H	5.13691	-0.31050	11.00124
H	-2.75720	3.88337	7.15539
H	-1.17670	1.14587	7.15539
H	-3.55681	2.42700	11.00124
H	-2.83775	1.18157	11.00124

Table 4: 3×3 MoS_2-WS_2

Atom	X	Y	Z
W	1.59000	0.91799	3.12500
W	0.00000	3.67195	3.12500
W	-1.59000	6.42591	3.12500
W	4.77000	0.91799	3.12500
W	3.18000	3.67195	3.12500
W	1.59000	6.42591	3.12500
W	7.95000	0.91799	3.12500
W	6.36000	3.67195	3.12500
W	4.77000	6.42591	3.12500
S	0.00000	1.83597	4.68750
S	-1.59000	4.58993	4.68750
S	-3.18000	7.34390	4.68750
S	3.18000	1.83597	4.68750
S	1.59000	4.58993	4.68750
S	0.00000	7.34390	4.68750
S	6.36000	1.83597	4.68750
S	4.77000	4.58993	4.68750
S	3.18000	7.34390	4.68750
S	0.00000	1.83597	1.56250
S	-1.59000	4.58993	1.56250
S	-3.18000	7.34390	1.56250
S	3.18000	1.83597	1.56250
S	1.59000	4.58993	1.56250
S	0.00000	7.34390	1.56250
S	6.36000	1.83597	1.56250
S	4.77000	4.58993	1.56250
S	3.18000	7.34390	1.56250
S	1.59000	-0.91799	1.56250
S	1.59000	-0.91799	4.68750
S	4.77000	-0.91799	1.56250
S	4.77000	-0.91799	4.68750
S	7.95000	-0.91799	1.56250
S	7.95000	-0.91799	4.68750
S	9.54000	1.83597	1.56250
S	9.54000	1.83597	4.68750
S	7.95000	4.58993	1.56250
S	7.95000	4.58993	4.68750
S	6.36000	7.34390	1.56250
S	6.36000	7.34390	4.68750
S	3.14712	9.13301	11.44493
S	3.14712	9.13301	8.43263
Mo	3.14712	7.30801	9.93873
S	4.72762	6.39551	8.43263
S	4.72762	6.39551	11.44493
S	-0.01388	9.13301	11.44493
S	-0.01388	9.13301	8.43263
Mo	-0.01388	7.30801	9.93873
Mo	4.72762	4.57051	9.93873
S	6.30812	3.65801	8.43263
S	-3.17488	9.13301	8.43263
S	-3.17488	9.13301	11.44493
S	6.30812	3.65801	11.44493
S	1.56662	6.39551	8.43263
S	1.56662	6.39551	11.44493
Mo	-3.17488	7.30801	9.93873
Mo	1.56662	4.57051	9.93873
Mo	6.30812	1.83301	9.93873
S	3.14712	3.65801	8.43263
S	-1.59438	6.39551	11.44493
S	-1.59438	6.39551	8.43263
S	7.88862	0.92051	8.43263
S	7.88862	0.92051	11.44493
S	3.14712	3.65801	11.44493
Mo	3.14712	1.83301	9.93873
Mo	-1.59438	4.57051	9.93873
S	-4.75538	6.39551	11.44493
S	-0.01388	3.65801	11.44493
S	-4.75538	6.39551	8.43263
S	4.72762	0.92051	11.44493
S	-0.01388	3.65801	8.43263
S	4.72762	0.92051	8.43263
Mo	-0.01388	1.83301	9.93873
S	-3.17488	3.65801	11.44493
S	-3.17488	3.65801	8.43263
S	1.56662	0.92051	8.43263
S	1.56662	0.92051	11.44493
S	-1.59438	0.92051	11.44493
S	-1.59438	0.92051	8.43263
H	-4.44281	7.08815	4.25218
H	-3.82721	6.33089	0.92648
H	0.38910	-0.97198	0.92648
H	0.73710	-1.88374	4.25218
H	3.56910	-0.97198	0.92648
H	3.91710	-1.88374	4.25218
H	6.74910	-0.97198	0.92648
H	7.09710	-1.88374	4.25218
H	9.11929	1.59307	0.29222
H	10.80281	1.58022	4.25218
H	7.52929	4.34703	0.29222
H	8.35992	5.81143	4.25219
H	5.93929	7.10100	0.29222
H	7.62281	7.08815	4.25218
H	4.00857	10.08718	11.00100
H	3.14712	8.66651	7.15514
H	-0.87533	10.08718	11.00100
H	-1.21739	9.20111	7.80293
H	-4.37839	9.20111	7.80293
H	-4.03633	10.08718	11.00100
H	7.34584	-0.15581	7.80293
H	8.28423	-0.30261	11.00099
H	-6.01243	6.66446	11.00099
H	-5.41611	7.40373	7.80293
H	-3.57049	2.43489	11.00099
H	-2.77088	3.89126	7.15514
H	-2.85143	1.18946	11.00099
H	-1.19038	1.15376	7.15514

Table 5: 2×2 $MoSe_2$-WS_2

Atom	X	Y	Z
Mo	0.00000	1.89833	3.23000
Mo	-1.64400	4.74582	3.23000
Mo	3.28800	1.89833	3.23000
Mo	1.64400	4.74582	3.23000
Se	1.64400	0.94916	4.89668
Se	0.00000	3.79665	4.89668
Se	4.93200	0.94916	4.89668
Se	3.28800	3.79665	4.89668
Se	1.64400	0.94916	1.56332
Se	0.00000	3.79665	1.56332
Se	4.93200	0.94916	1.56332
Se	3.28800	3.79665	1.56332
Se	-1.64400	0.94916	1.56332
Se	-1.64400	0.94916	4.89668
Se	-3.28800	3.79665	1.56332
Se	-3.28800	3.79665	4.89668
Se	-1.64400	6.64415	1.56332
Se	-1.64400	6.64415	4.89668
Se	1.64400	6.64415	1.56332
Se	1.64400	6.64415	4.89668
S	1.70467	-0.84065	8.64082
S	1.70467	-0.84065	11.76582
W	1.70467	0.99535	10.20332
S	0.11467	1.91335	11.76582
S	0.11467	1.91335	8.64082
S	4.88467	-0.84065	8.64082
S	4.88467	-0.84065	11.76582
W	0.11467	3.74925	10.20332
W	4.88467	0.99535	10.20332
S	3.29467	1.91335	8.64082

Atom	X	Y	Z
S	-1.47533	4.66725	11.76582
S	-1.47533	4.66725	8.64082
S	3.29467	1.91335	11.76582
W	3.29467	3.74925	10.20332
S	6.47467	1.91335	11.76582
S	6.47467	1.91335	8.64082
S	1.70467	4.66725	8.64082
S	1.70467	4.66725	11.76582
S	4.88467	4.66725	8.64082
S	4.88467	4.66725	11.76582
H	5.40295	-0.40530	4.42367
H	4.30481	-0.22493	0.85042
H	-1.15812	1.22968	0.16142
H	-2.11495	-0.40530	4.42367
H	-2.80212	4.07718	0.16142
H	-3.75895	2.44219	4.42367
H	-0.31361	6.68803	0.85042
H	-0.70648	7.72923	4.42367
H	1.64400	6.08311	0.16142
H	0.70648	7.72923	4.42367
H	0.50377	-0.89465	8.00480
H	0.85178	-1.80640	11.33049
H	3.68377	-0.89465	8.00480
H	4.03178	-1.80640	11.33049
H	-1.88525	5.88875	11.33049
H	-0.92162	5.73424	8.00478
H	7.73748	1.65759	11.33051
H	6.05397	1.67045	7.37054
H	4.33096	5.73424	8.00478
H	5.29459	5.88875	11.33049

Table 6: 3×3 $MoSe_2$-WS_2

Atom	X	Y	Z
Mo	0.00000	1.89833	4.07000
Mo	-1.64400	4.74582	4.07000
Mo	-3.28800	7.59331	4.07000
Mo	3.28800	1.89833	4.07000
Mo	1.64400	4.74582	4.07000
Mo	0.00000	7.59331	4.07000
Mo	6.57600	1.89833	4.07000
Mo	4.93200	4.74582	4.07000
Mo	3.28800	7.59331	4.07000
Se	1.64400	0.94916	5.73668
Se	0.00000	3.79665	5.73668
Se	-1.64400	6.64415	5.73668
Se	4.93200	0.94916	5.73668
Se	3.28800	3.79665	5.73668
Se	1.64400	6.64415	5.73668
Se	8.22000	0.94916	5.73668
Se	6.57600	3.79665	5.73668
Se	4.93200	6.64415	5.73668
Se	1.64400	0.94916	2.40332
Se	0.00000	3.79665	2.40332
Se	-1.64400	6.64415	2.40332
Se	4.93200	0.94916	2.40332
Se	3.28800	3.79665	2.40332
Se	1.64400	6.64415	2.40332
Se	8.22000	0.94916	2.40332
Se	6.57600	3.79665	2.40332
Se	4.93200	6.64415	2.40332
Se	-1.64400	0.94916	2.40332
Se	-1.64400	0.94916	5.73668
Se	-3.28800	3.79665	2.40332
Se	-3.28800	3.79665	5.73668
Se	-4.93200	6.64415	2.40332
Se	-4.93200	6.64415	5.73668
Se	-3.28800	9.49164	2.40332
Se	-3.28800	9.49164	5.73668
Se	0.00000	9.49164	2.40332
Se	0.00000	9.49164	5.73668
Se	3.28800	9.49164	2.40332
Se	3.28800	9.49164	5.73668
S	6.38877	7.32729	12.60634
S	6.38877	7.32729	9.48134
W	4.79877	6.40929	11.04384
S	3.20877	7.32729	9.48134
S	3.20877	7.32729	12.60634
S	7.97877	4.57329	9.48134
S	7.97877	4.57329	12.60634
W	1.61877	6.40929	11.04384
W	6.38877	3.65529	11.04384
S	9.56877	1.81939	12.60634
S	9.56877	1.81939	9.48134
S	0.02877	7.32729	12.60634
S	0.02877	7.32729	9.48134
S	4.79877	4.57329	12.60634
S	4.79877	4.57329	9.48134
W	-1.56123	6.40929	11.04384
W	3.20877	3.65529	11.04384
W	7.97877	0.90139	11.04384
S	6.38877	1.81939	9.48134
S	-3.15123	7.32729	12.60634
S	6.38877	1.81939	12.60634
S	-3.15123	7.32729	9.48134
S	1.61877	4.57329	9.48134
S	1.61877	4.57329	12.60634
W	4.79877	0.90139	11.04384
W	0.02877	3.65529	11.04384
S	7.97877	-0.93461	12.60634
S	-1.56123	4.57329	9.48134
S	3.20877	1.81939	12.60634
S	-1.56123	4.57329	12.60634
S	3.20877	1.81939	9.48134
S	7.97877	-0.93461	9.48134
W	1.61877	0.90139	11.04384
S	0.02877	1.81939	12.60634
S	0.02877	1.81939	9.48134
S	4.79877	-0.93461	12.60634
S	4.79877	-0.93461	9.48134
S	1.61877	-0.93461	9.48134
S	1.61877	-0.93461	12.60634
H	9.62847	1.21854	5.26367
H	8.92320	2.07937	1.69042
H	-2.34720	2.07937	1.69042
H	-3.05247	1.21854	5.26367
H	-3.99120	4.92686	1.69042
H	-4.69647	4.06603	5.26367
H	-5.63520	7.77436	1.69042
H	-6.34047	6.91353	5.26367
H	-4.61839	9.53552	1.69042
H	-4.22552	10.57672	5.26367
H	1.33039	9.53552	1.69042
H	0.93752	10.57672	5.26367
H	4.61839	9.53552	1.69042
H	4.22552	10.57672	5.26367
H	6.79868	8.54880	12.17102
H	5.96807	7.08439	8.21106
H	8.62598	3.56028	8.84532
H	9.24158	4.31753	12.17103
H	10.83158	1.56363	12.17103
H	10.21598	0.80638	8.84532
H	-3.56114	8.54880	12.17102
H	-2.73053	7.08439	8.21106
H	8.83166	-1.90036	12.17101
H	9.17967	-0.98861	8.84532
H	3.94588	-1.90036	12.17101
H	4.79877	-0.44883	8.21106
H	1.61877	-0.44883	8.21106
H	2.47166	-1.90036	12.17101

Table 7: 2×2 $MoSe_2$-$MoSe_2$

Atom	X	Y	Z
Mo	0.00000	1.89833	3.23000
Mo	-1.64400	4.74582	3.23000
Mo	3.28800	1.89833	3.23000
Mo	1.64400	4.74582	3.23000
Mo	1.64400	0.94916	9.69000
Mo	0.00000	3.79665	9.69000
Mo	4.93200	0.94916	9.69000
Mo	3.28800	3.79665	9.69000
Se	0.00000	1.89833	8.02332
Se	-1.64400	4.74582	8.02332
Se	3.28800	1.89833	8.02332
Se	1.64400	4.74582	8.02332
Se	1.64400	0.94916	4.89668
Se	0.00000	3.79665	4.89668
Se	4.93200	0.94916	4.89668
Se	3.28800	3.79665	4.89668
Se	1.64400	0.94916	1.56332
Se	0.00000	3.79665	1.56332
Se	4.93200	0.94916	1.56332
Se	3.28800	3.79665	1.56332
Se	0.00000	1.89833	11.35668
Se	-1.64400	4.74582	11.35668
Se	3.28800	1.89833	11.35668
Se	1.64400	4.74582	11.35668
Se	-1.64400	0.94916	1.56332
Se	-1.64400	0.94916	4.89668
Se	-3.28800	3.79665	1.56332
Se	-3.28800	3.79665	4.89668
Se	-1.64400	6.64415	1.56332
Se	-1.64400	6.64415	4.89668

Atom	X	Y	Z
Se	1.64400	6.64415	1.56332
Se	1.64400	6.64415	4.89668
Se	1.64400	-0.94916	8.02332
Se	1.64400	-0.94916	11.35668
Se	4.93200	-0.94916	8.02332
Se	4.93200	-0.94916	11.35668
Se	6.57600	1.89833	8.02332
Se	6.57600	1.89833	11.35668
Se	4.93200	4.74582	8.02332
Se	4.93200	4.74582	11.35668
H	-1.15812	4.46530	6.62142
H	5.40295	-0.40530	4.42367
H	4.30481	-0.22493	0.85042
H	-3.05247	4.47644	10.88367
H	-1.01681	-0.22493	0.85042
H	-2.11495	-0.40530	4.42367
H	-3.99120	4.92687	0.85042
H	-4.69647	4.06603	4.42367
H	-2.97439	6.68803	0.85042
H	-2.58152	7.72923	4.42367
H	0.31361	6.68803	0.85042
H	0.70648	7.72923	4.42367
H	1.64400	-0.38812	6.62142
H	0.70648	-2.03425	10.88367
H	4.93200	-0.38812	6.62142
H	3.99448	-2.03425	10.88367
H	5.94881	3.07242	7.31042
H	7.04695	3.25279	10.88367
H	4.44612	4.46530	6.62142
H	6.34047	4.47644	10.88367

Table 8: 3×3 $MoSe_2$-$MoSe_2$

Atom	X	Y	Z
Mo	0.00000	1.89833	29.07000
Mo	-1.64400	4.74582	29.07000
Mo	-3.28800	7.59331	29.07000
Mo	3.28800	1.89833	29.07000
Mo	1.64400	4.74582	29.07000
Mo	0.00000	7.59331	29.07000
Mo	6.57600	1.89833	29.07000
Mo	4.93200	4.74582	29.07000
Mo	3.28800	7.59331	29.07000
Mo	1.64400	0.94916	35.53000
Mo	0.00000	3.79665	35.53000
Mo	-1.64400	6.64415	35.53000
Mo	4.93200	0.94916	35.53000
Mo	3.28800	3.79665	35.53000
Mo	1.64400	6.64415	35.53000
Mo	8.22000	0.94916	35.53000
Mo	6.57600	3.79665	35.53000
Mo	4.93200	6.64415	35.53000
Se	0.00000	1.89833	33.86332
Se	-1.64400	4.74582	33.86332
Se	-3.28800	7.59331	33.86332
Se	3.28800	1.89833	33.86332
Se	1.64400	4.74582	33.86332
Se	0.00000	7.59331	33.86332
Se	6.57600	1.89833	33.86332
Se	4.93200	4.74582	33.86332
Se	3.28800	7.59331	33.86332
Se	1.64400	0.94916	30.73668
Se	0.00000	3.79665	30.73668
Se	-1.64400	6.64415	30.73668
Se	4.93200	0.94916	30.73668
Se	3.28800	3.79665	30.73668
Se	1.64400	6.64415	30.73668
Se	8.22000	0.94916	30.73668
Se	6.57600	3.79665	30.73668
Se	4.93200	6.64415	30.73668
Se	1.64400	0.94916	27.40332
Se	0.00000	3.79665	27.40332
Se	-1.64400	6.64415	27.40332
Se	4.93200	0.94916	27.40332
Se	3.28800	3.79665	27.40332
Se	1.64400	6.64415	27.40332
Se	8.22000	0.94916	27.40332
Se	6.57600	3.79665	27.40332
Se	4.93200	6.64415	27.40332
Se	0.00000	1.89833	37.19668
Se	-1.64400	4.74582	37.19668
Se	-3.28800	7.59331	37.19668
Se	3.28800	1.89833	37.19668
Se	1.64400	4.74582	37.19668
Se	0.00000	7.59331	37.19668
Se	6.57600	1.89833	37.19668
Se	4.93200	4.74582	37.19668

Atom	X	Y	Z
Se	3.28800	7.59331	37.19668
Se	-1.64400	0.94916	27.40332
Se	-1.64400	0.94916	30.73668
Se	-3.28800	3.79665	27.40332
Se	-3.28800	3.79665	30.73668
Se	-4.93200	6.64415	27.40332
Se	-4.93200	6.64415	30.73668
Se	-3.28800	9.49164	27.40332
Se	-3.28800	9.49164	30.73668
Se	0.00000	9.49164	27.40332
Se	0.00000	9.49164	30.73668
Se	3.28800	9.49164	27.40332
Se	3.28800	9.49164	30.73668
Se	1.64400	-0.94916	33.86332
Se	1.64400	-0.94916	37.19668
Se	4.93200	-0.94916	33.86332
Se	4.93200	-0.94916	37.19668
Se	8.22000	-0.94916	33.86332
Se	8.22000	-0.94916	37.19668
Se	9.86400	1.89833	33.86332
Se	9.86400	1.89833	37.19668
Se	8.22000	4.74582	33.86332
Se	8.22000	4.74582	37.19668
Se	6.57600	7.59331	33.86332
Se	6.57600	7.59331	37.19668
H	-2.80212	7.31279	32.46142
H	9.62847	1.21854	30.26367
H	8.92320	2.07938	26.69042
H	-3.75895	8.94777	36.72367
H	-1.01681	-0.22493	26.69042
H	-2.11495	-0.40530	30.26367
H	-3.99120	4.92687	26.69042
H	-4.69647	4.06603	30.26367
H	-5.63520	7.77436	26.69042
H	-6.34047	6.91352	30.26367
H	-4.61839	9.53552	26.69042
H	-4.22552	10.57672	30.26367
H	-1.33039	9.53552	26.69042
H	-0.93752	10.57672	30.26367
H	4.61839	9.53552	26.69042
H	4.22552	10.57672	30.26367
H	0.31361	-0.99305	33.15042
H	0.70648	-2.03425	36.72367
H	3.60161	-0.99305	33.15042
H	3.99448	-2.03425	36.72367
H	6.88961	-0.99305	33.15042
H	7.28248	-2.03425	36.72367
H	9.37812	1.61781	32.46142
H	10.33495	3.25279	36.72367
H	8.92320	3.61561	33.15042
H	9.62847	4.47644	36.72367
H	6.09012	7.31279	32.46142
H	7.98447	7.32393	36.72367

Table 9: 2×2 WSe_2-WSe_2

Atom	X	Y	Z
W	1.64250	0.94830	3.24600
W	0.00000	3.79319	3.24600
W	4.92750	0.94830	3.24600
W	3.28500	3.79319	3.24600
W	-0.00000	1.89660	9.73800
W	-1.64250	4.74149	9.73800
W	3.28500	1.89660	9.73800
W	1.64250	4.74149	9.73800
Se	0.00000	1.89660	1.55808
Se	-1.64250	4.74149	1.55808
Se	3.28500	1.89660	1.55808
Se	1.64250	4.74149	1.55808
Se	1.64250	0.94830	11.42592
Se	0.00000	3.79319	11.42592
Se	4.92750	0.94830	11.42592
Se	3.28500	3.79319	11.42592
Se	1.64250	0.94830	8.05008
Se	0.00000	3.79319	8.05008
Se	4.92750	0.94830	8.05008
Se	3.28500	3.79319	8.05008
Se	0.00000	1.89660	4.93392
Se	-1.64250	4.74149	4.93392
Se	3.28500	1.89660	4.93392
Se	1.64250	4.74149	4.93392
Se	1.64250	-0.94830	1.55808
Se	1.64250	-0.94830	4.93392
Se	4.92750	-0.94830	1.55808
Se	4.92750	-0.94830	4.93392
Se	6.57000	1.89660	1.55808
Se	6.57000	1.89660	4.93392

Atom	X	Y	Z
Se	4.92750	4.74149	1.55808
Se	4.92750	4.74149	4.93392
Se	-1.64250	0.94830	8.05008
Se	-1.64250	0.94830	11.42592
Se	-3.28500	3.79319	8.05008
Se	-3.28500	3.79319	11.42592
Se	-1.64250	6.63809	8.05008
Se	-1.64250	6.63809	11.42592
Se	1.64250	6.63809	8.05008
Se	1.64250	6.63809	11.42592
H	-1.14846	4.45625	0.15999
H	6.33872	1.21141	10.95759
H	5.62395	2.08092	7.33437
H	-3.05372	4.47837	4.46559
H	1.64250	-0.37783	0.15999
H	2.57597	-2.03889	4.46559
H	3.59840	-0.98513	0.84237
H	3.99403	-2.03889	4.46559
H	6.07596	1.61136	0.15999
H	7.04775	3.25030	4.46559
H	4.43346	4.45625	0.15999
H	6.33872	4.47837	4.46559
H	-1.00985	-0.22115	7.33437
H	-2.12025	-0.40541	10.95759
H	-2.79096	4.07843	6.65199
H	-3.76275	2.43948	10.95759
H	-0.31340	6.67492	7.33437
H	-0.70903	7.72868	10.95759
H	2.97160	6.67492	7.33437
H	2.57597	7.72868	10.95759

Table 10: 3×3 WSe_2-WSe_2

Atom	X	Y	Z
W	1.64250	0.94830	3.24600
W	0.00000	3.79319	3.24600
W	-1.64250	6.63809	3.24600
W	4.92750	0.94830	3.24600
W	3.28500	3.79319	3.24600
W	1.64250	6.63809	3.24600
W	8.21250	0.94830	3.24600
W	6.57000	3.79319	3.24600
W	4.92750	6.63809	3.24600
W	-0.00000	1.89660	9.73800
W	-1.64250	4.74149	9.73800
W	-3.28500	7.58638	9.73800
W	3.28500	1.89660	9.73800
W	1.64250	4.74149	9.73800
W	-0.00000	7.58638	9.73800
W	6.57000	1.89660	9.73800
W	4.92750	4.74149	9.73800
W	3.28500	7.58638	9.73800
Se	0.00000	1.89660	1.55808
Se	-1.64250	4.74149	1.55808
Se	-3.28500	7.58638	1.55808
Se	3.28500	1.89660	1.55808
Se	1.64250	4.74149	1.55808
Se	0.00000	7.58638	1.55808
Se	6.57000	1.89660	1.55808
Se	4.92750	4.74149	1.55808
Se	3.28500	7.58638	1.55808
Se	1.64250	0.94830	11.42592
Se	0.00000	3.79319	11.42592
Se	-1.64250	6.63809	11.42592
Se	4.92750	0.94830	11.42592
Se	3.28500	3.79319	11.42592
Se	1.64250	6.63809	11.42592
Se	8.21250	0.94830	11.42592
Se	6.57000	3.79319	11.42592
Se	4.92750	6.63809	11.42592
Se	1.64250	0.94830	8.05008
Se	0.00000	3.79319	8.05008
Se	-1.64250	6.63809	8.05008
Se	4.92750	0.94830	8.05008
Se	3.28500	3.79319	8.05008
Se	1.64250	6.63809	8.05008
Se	8.21250	0.94830	8.05008
Se	6.57000	3.79319	8.05008
Se	4.92750	6.63809	8.05008
Se	0.00000	1.89660	4.93392
Se	-1.64250	4.74149	4.93392
Se	-3.28500	7.58638	4.93392
Se	3.28500	1.89660	4.93392
Se	1.64250	4.74149	4.93392
Se	0.00000	7.58638	4.93392
Se	6.57000	1.89660	4.93392
Se	4.92750	4.74149	4.93392
Se	3.28500	7.58638	4.93392
Se	1.64250	-0.94830	1.55808
Se	1.64250	-0.94830	4.93392
Se	4.92750	-0.94830	1.55808
Se	4.92750	-0.94830	4.93392
Se	8.21250	-0.94830	1.55808
Se	8.21250	-0.94830	4.93392
Se	9.85500	1.89660	1.55808
Se	9.85500	1.89660	4.93392
Se	8.21250	4.74149	1.55808
Se	8.21250	4.74149	4.93392
Se	6.57000	7.58638	1.55808
Se	6.57000	7.58638	4.93392
Se	-1.64250	0.94830	8.05008
Se	-1.64250	0.94830	11.42592
Se	-3.28500	3.79319	8.05008
Se	-3.28500	3.79319	11.42592
Se	-4.92750	6.63809	8.05008
Se	-4.92750	6.63809	11.42592
Se	-3.28500	9.48298	8.05008
Se	-3.28500	9.48298	11.42592
Se	0.00000	9.48298	8.05008
Se	0.00000	9.48298	11.42592
Se	3.28500	9.48298	8.05008
Se	3.28500	9.48298	11.42592
H	-2.65235	8.75583	0.84237
H	8.69025	-0.40541	10.95759
H	7.71846	1.23353	6.65199
H	-3.76275	8.94009	4.46559
H	1.64250	-0.37783	0.15999
H	2.57597	-2.03889	4.46559
H	4.92750	-0.37783	0.15999
H	3.99403	-2.03889	4.46559
H	6.88340	-0.98513	0.84237
H	7.27903	-2.03889	4.46559
H	9.36096	1.61136	0.15999
H	10.33275	3.25030	4.46559
H	7.71846	4.45625	0.15999
H	9.62372	4.47837	4.46559
H	7.26645	6.45376	0.84237
H	7.98122	7.32327	4.46559
H	-1.14846	1.23353	6.65199
H	-3.05372	1.21141	10.95759
H	-2.79096	4.07843	6.65199
H	-2.79096	4.07843	12.82401
H	-4.43346	6.92332	6.65199
H	-6.33872	6.90120	10.95759
H	-1.95590	9.51981	7.33437
H	-2.35153	10.57357	10.95759
H	1.32910	9.51981	7.33437
H	0.93347	10.57357	10.95759
H	3.28500	8.91251	6.65199
H	2.35153	10.57357	10.95759

Table 11: 2×2 MoS_2-MoS_2

Atom	X	Y	Z
Mo	0.00000	1.82500	3.07375
Mo	-1.58050	4.56251	3.07375
Mo	3.16100	1.82500	3.07375
Mo	1.58050	4.56251	3.07375
Mo	1.58050	0.91250	9.22125
Mo	0.00000	3.65001	9.22125
Mo	4.74150	0.91250	9.22125
Mo	3.16100	3.65001	9.22125
S	0.00000	1.82500	7.71511
S	-1.58050	4.56251	7.71511
S	3.16100	1.82500	7.71511
S	1.58050	4.56251	7.71511
S	1.58050	0.91250	4.57989
S	0.00000	3.65001	4.57989
S	4.74150	0.91250	4.57989
S	3.16100	3.65001	4.57989
S	1.58050	0.91250	1.56761
S	0.00000	3.65001	1.56761
S	4.74150	0.91250	1.56761
S	3.16100	3.65001	1.56761
S	0.00000	1.82500	10.72739
S	-1.58050	4.56251	10.72739
S	3.16100	1.82500	10.72739
S	1.58050	4.56251	10.72739
S	-1.58050	0.91250	1.56761
S	-1.58050	0.91250	4.57989
S	-3.16100	3.65001	1.56761
S	-3.16100	3.65001	4.57989
S	-1.58050	6.38751	1.56761
S	-1.58050	6.38751	4.57989

Atom	X	Y	Z
S	1.58050	6.38751	1.56761
S	1.58050	6.38751	4.57989
S	1.58050	-0.91250	7.71511
S	1.58050	-0.91250	10.72739
S	4.74150	-0.91250	7.71511
S	4.74150	-0.91250	10.72739
S	6.32200	1.82500	7.71511
S	6.32200	1.82500	10.72739
S	4.74150	4.56251	7.71511
S	4.74150	4.56251	10.72739
H	-1.17649	4.32926	6.43763
H	5.13709	-0.31062	4.13594
H	4.19873	-0.16384	0.93794
H	-2.83755	4.29354	10.28344
H	-1.03773	-0.16384	0.93794
H	-1.97609	-0.31062	4.13594
H	-3.82176	4.65823	0.93794
H	-4.41805	3.91898	4.13594
H	-2.78403	6.45563	0.93794
H	-2.44196	7.34167	4.13594
H	0.37697	6.45563	0.93794
H	0.71904	7.34167	4.13594
H	1.58050	-0.44599	6.43763
H	0.71904	-1.86665	10.28344
H	4.74150	-0.44599	6.43763
H	5.60296	-1.86665	10.28344
H	5.77923	2.90135	7.08544
H	6.71759	3.04812	10.28344
H	4.33749	4.32926	6.43763
H	5.99855	4.29354	10.28344

Table 12: 3×3 MoS_2-MoS_2

Atom	X	Y	Z
Mo	0.00000	1.82500	27.66375
Mo	-1.58050	4.56251	27.66375
Mo	-3.16100	7.30002	27.66375
Mo	3.16100	1.82500	27.66375
Mo	1.58050	4.56251	27.66375
Mo	0.00000	7.30002	27.66375
Mo	6.32200	1.82500	27.66375
Mo	4.74150	4.56251	27.66375
Mo	3.16100	7.30002	27.66375
Mo	1.58050	0.91250	33.81125
Mo	0.00000	3.65001	33.81125
Mo	-1.58050	6.38751	33.81125
Mo	4.74150	0.91250	33.81125
Mo	3.16100	3.65001	33.81125
Mo	1.58050	6.38751	33.81125
Mo	7.90250	0.91250	33.81125
Mo	6.32200	3.65001	33.81125
Mo	4.74150	6.38751	33.81125
S	0.00000	1.82500	32.30511
S	-1.58050	4.56251	32.30511
S	-3.16100	7.30002	32.30511
S	3.16100	1.82500	32.30511
S	1.58050	4.56251	32.30511
S	0.00000	7.30002	32.30511
S	6.32200	1.82500	32.30511
S	4.74150	4.56251	32.30511
S	3.16100	7.30002	32.30511
S	1.58050	0.91250	29.16989
S	0.00000	3.65001	29.16989
S	-1.58050	6.38751	29.16989
S	4.74150	0.91250	29.16989
S	3.16100	3.65001	29.16989
S	1.58050	6.38751	29.16989
S	7.90250	0.91250	29.16989
S	6.32200	3.65001	29.16989
S	4.74150	6.38751	29.16989
S	1.58050	0.91250	26.15761
S	0.00000	3.65001	26.15761
S	-1.58050	6.38751	26.15761
S	4.74150	0.91250	26.15761
S	3.16100	3.65001	26.15761
S	1.58050	6.38751	26.15761
S	7.90250	0.91250	26.15761
S	6.32200	3.65001	26.15761
S	4.74150	6.38751	26.15761
S	0.00000	1.82500	35.31739
S	-1.58050	4.56251	35.31739
S	-3.16100	7.30002	35.31739
S	3.16100	1.82500	35.31739
S	1.58050	4.56251	35.31739
S	0.00000	7.30002	35.31739
S	6.32200	1.82500	35.31739
S	4.74150	4.56251	35.31739
S	3.16100	7.30002	35.31739
S	-1.58050	0.91250	26.15761
S	-1.58050	0.91250	29.16989
S	-3.16100	3.65001	26.15761
S	-3.16100	3.65001	29.16989
S	-4.74150	6.38751	26.15761
S	-4.74150	6.38751	29.16989
S	-3.16100	9.12502	26.15761
S	-3.16100	9.12502	29.16989
S	0.00000	9.12502	26.15761
S	0.00000	9.12502	29.16989
S	3.16100	9.12502	26.15761
S	3.16100	9.12502	29.16989
S	1.58050	-0.91250	32.30511
S	1.58050	-0.91250	35.31739
S	4.74150	-0.91250	32.30511
S	4.74150	-0.91250	35.31739
S	7.90250	-0.91250	32.30511
S	7.90250	-0.91250	35.31739
S	9.48300	1.82500	32.30511
S	9.48300	1.82500	35.31739
S	7.90250	4.56251	32.30511
S	7.90250	4.56251	35.31739
S	6.32200	7.30002	32.30511
S	6.32200	7.30002	35.31739
H	-2.75699	7.06676	31.02763
H	9.15955	1.18147	28.72594
H	8.56326	1.92073	25.52794
H	-3.55659	8.52314	34.87344
H	-1.03773	-0.16384	25.52794
H	-1.97609	-0.31062	28.72594
H	-2.61823	2.57366	25.52794
H	-3.55659	2.42689	28.72594
H	-5.40226	7.39574	25.52794
H	-5.99855	6.65648	28.72594
H	-4.36453	9.19314	25.52794
H	-4.02246	10.07917	28.72594
H	-1.20353	9.19314	25.52794
H	-0.86146	10.07917	28.72594
H	4.36453	9.19314	25.52794
H	4.02246	10.07917	28.72594
H	0.37697	-0.98062	31.67544
H	0.71904	-1.86665	34.87344
H	5.94503	-0.98062	31.67544
H	5.60296	-1.86665	34.87344
H	9.10603	-0.98062	31.67544
H	8.76396	-1.86665	34.87344
H	10.14376	0.81678	31.67544
H	10.74005	1.55604	34.87344
H	8.56326	3.55429	31.67544
H	9.15955	4.29354	34.87344
H	5.91799	7.06676	31.02763
H	6.71759	8.52314	34.87344

Table 13: 2×2 WS_2-WS_2

Atom	X	Y	Z
W	1.59000	0.91799	3.12500
W	0.00000	3.67195	3.12500
W	4.77000	0.91799	3.12500
W	3.18000	3.67195	3.12500
W	-0.00000	1.83597	9.37500
W	-1.59000	4.58993	9.37500
W	3.18000	1.83597	9.37500
W	1.59000	4.58993	9.37500
S	0.00000	1.83597	4.68750
S	-1.59000	4.58993	4.68750
S	3.18000	1.83597	4.68750
S	1.59000	4.58993	4.68750
S	1.59000	0.91799	7.81250
S	0.00000	3.67195	7.81250
S	4.77000	0.91799	7.81250
S	3.18000	3.67195	7.81250
S	1.59000	0.91799	10.93750
S	0.00000	3.67195	10.93750
S	4.77000	0.91799	10.93750
S	3.18000	3.67195	10.93750
S	0.00000	1.83597	1.56250
S	-1.59000	4.58993	1.56250
S	3.18000	1.83597	1.56250
S	1.59000	4.58993	1.56250
S	1.59000	-0.91799	1.56250
S	1.59000	-0.91799	4.68750
S	4.77000	-0.91799	1.56250
S	4.77000	-0.91799	4.68750
S	6.36000	1.83597	1.56250
S	6.36000	1.83597	4.68750

Atom	X	Y	Z
S	4.77000	4.58993	1.56250
S	4.77000	4.58993	4.68750
S	-1.59000	0.91799	7.81250
S	-1.59000	0.91799	10.93750
S	-3.18000	3.67195	7.81250
S	-3.18000	3.67195	10.93750
S	-1.59000	6.42591	7.81250
S	-1.59000	6.42591	10.93750
S	1.59000	6.42591	7.81250
S	1.59000	6.42591	10.93750
H	-2.85281	4.33418	4.25218
H	4.21631	-0.14902	7.17648
H	5.17992	-0.30352	10.50218
H	-1.16929	4.34704	0.29222
H	1.59000	-0.43219	0.29222
H	0.73711	-1.88374	4.25218
H	3.56910	-0.97198	0.92648
H	3.91711	-1.88374	4.25218
H	5.93929	1.59308	0.29222
H	6.76992	3.05748	4.25218
H	4.34929	4.34704	0.29222
H	5.17992	5.81144	4.25218
H	-1.16929	1.16088	6.54222
H	-2.85281	1.17374	10.50218
H	-2.62631	2.60494	7.17648
H	-3.58992	2.45045	10.50218
H	-1.59000	5.94012	6.54222
H	-2.44289	7.39166	10.50218
H	1.59000	5.94012	6.54222
H	0.73711	7.39166	10.50218

Table 14: 3×3 WS_2-WS_2

Atom	X	Y	Z
W	1.59000	0.91799	28.12500
W	0.00000	3.67195	28.12500
W	-1.59000	6.42591	28.12500
W	4.77000	0.91799	28.12500
W	3.18000	3.67195	28.12500
W	1.59000	6.42591	28.12500
W	7.95000	0.91799	28.12500
W	6.36000	3.67195	28.12500
W	4.77000	6.42591	28.12500
W	-0.00000	1.83597	34.37500
W	-1.59000	4.58993	34.37500
W	-3.18000	7.34390	34.37500
W	3.18000	1.83597	34.37500
W	1.59000	4.58993	34.37500
W	-0.00000	7.34390	34.37500
W	6.36000	1.83597	34.37500
W	4.77000	4.58993	34.37500
W	3.18000	7.34390	34.37500
S	0.00000	1.83597	29.68750
S	-1.59000	4.58993	29.68750
S	-3.18000	7.34390	29.68750
S	3.18000	1.83597	29.68750
S	1.59000	4.58993	29.68750
S	0.00000	7.34390	29.68750
S	6.36000	1.83597	29.68750
S	4.77000	4.58993	29.68750
S	3.18000	7.34390	29.68750
S	1.59000	0.91799	32.81250
S	0.00000	3.67195	32.81250
S	-1.59000	6.42591	32.81250
S	4.77000	0.91799	32.81250
S	3.18000	3.67195	32.81250
S	1.59000	6.42591	32.81250
S	7.95000	0.91799	32.81250
S	6.36000	3.67195	32.81250
S	4.77000	6.42591	32.81250
S	1.59000	0.91799	35.93750
S	0.00000	3.67195	35.93750
S	-1.59000	6.42591	35.93750
S	4.77000	0.91799	35.93750
S	3.18000	3.67195	35.93750
S	1.59000	6.42591	35.93750
S	7.95000	0.91799	35.93750
S	6.36000	3.67195	35.93750
S	4.77000	6.42591	35.93750
S	0.00000	1.83597	26.56250
S	-1.59000	4.58993	26.56250
S	-3.18000	7.34390	26.56250
S	3.18000	1.83597	26.56250
S	1.59000	4.58993	26.56250
S	0.00000	7.34390	26.56250
S	6.36000	1.83597	26.56250
S	4.77000	4.58993	26.56250

Atom	X	Y	Z
S	3.18000	7.34390	26.56250
S	1.59000	-0.91799	26.56250
S	1.59000	-0.91799	29.68750
S	4.77000	-0.91799	26.56250
S	4.77000	-0.91799	29.68750
S	7.95000	-0.91799	26.56250
S	7.95000	-0.91799	29.68750
S	9.54000	1.83597	26.56250
S	9.54000	1.83597	29.68750
S	7.95000	4.58993	26.56250
S	7.95000	4.58993	29.68750
S	6.36000	7.34390	26.56250
S	6.36000	7.34390	29.68750
S	-1.59000	0.91799	32.81250
S	-1.59000	0.91799	35.93750
S	-3.18000	3.67195	32.81250
S	-3.18000	3.67195	35.93750
S	-4.77000	6.42591	32.81250
S	-4.77000	6.42591	35.93750
S	-3.18000	9.17987	32.81250
S	-3.18000	9.17987	35.93750
S	0.00000	9.17987	32.81250
S	0.00000	9.17987	35.93750
S	3.18000	9.17987	32.81250
S	3.18000	9.17987	35.93750
H	-3.58992	8.56540	29.25218
H	8.59721	1.93100	32.17648
H	9.21281	1.17374	35.50218
H	-2.75929	7.10100	25.29222
H	0.38910	-0.97198	25.92648
H	0.73711	-1.88374	29.25218
H	4.77000	-0.43219	25.29222
H	5.62289	-1.88374	29.25218
H	7.95000	-0.43219	25.29222
H	8.80289	-1.88374	29.25218
H	8.98631	2.90298	25.92648
H	9.94992	3.05748	29.25218
H	7.52929	4.34704	25.29222
H	8.35992	5.81144	29.25218
H	5.93929	7.10100	25.29222
H	7.62281	7.08814	29.25218
H	-1.03631	-0.14902	32.17648
H	-1.99992	-0.30352	35.50218
H	-3.82721	4.68496	32.17648
H	-4.44281	3.92770	35.50218
H	-5.41721	7.43892	32.17648
H	-6.03281	6.68166	35.50218
H	-4.38090	9.23386	32.17648
H	-4.03289	10.14562	35.50218
H	1.20090	9.23386	32.17648
H	0.85289	10.14562	35.50218
H	3.18000	8.69408	31.54222
H	2.32711	10.14562	35.50218
